# Gastrointestinal Complications in Critically Ill Children: Experience from A Resource-Limited Country

**DOI:** 10.12669/pjms.37.3.3493

**Published:** 2021

**Authors:** Sidra Ishaque, Mariam Shakir, Asma Ladak, Anwar Ul Haque

**Affiliations:** 1 Dr. Sidra Ishaque, FCPS. Department of Pediatrics and Child Health, The Aga Khan University Hospital, Karachi, Pakistan; 2 Dr. Mariam Shakir, FCPS. Department of Pediatrics and Child Health, The Aga Khan University Hospital, Karachi, Pakistan; 3 Asma Ladak, MBBS. Medical College, The Aga Khan University Hospital, Karachi, Pakistan; 4 Dr. Anwar Ul Haque MD. Department of Pediatrics, Liaquat National Hospital, Karachi, Pakistan

**Keywords:** Gastrointestinal Complications, Critically Ill, Children

## Abstract

**Objectives:**

To determine the frequency and predictors of outcome of gastrointestinal complications (GIC) in critically ill children.

**Methods:**

This descriptive study was prospectively conducted in The Pediatric Intensive Care Unit (PICU), The Aga Khan University Hospital (AKUH), Karachi, from September 2015 to January 2017. After obtaining approval from the Ethical Review Committee of AKUH and informed consent from the parents, all children (aged one month to 18 years), of either gender, admitted to the Pediatric Intensive Care Unit (PICU) during the study period were included. The frequency of the defined GIC: vomiting, high gastric residue volume (GRV), diarrhea, constipation, and gastrointestinal bleed were recorded daily for the first week of the PICU stay. The data was collected by the primary investigator on a predesigned data collection form with inclusion of variables and predictors in light of existing literature and local expertise. The questionnaire was shared with the Pediatric Critical Care Medicine faculty and a consensus was sought on the elements to be incorporated.

**Results:**

GIC developed within the first 48 hours of admission in 78 (41%) patients. Of the patients who developed GIC, 37 (47.4%) patients developed high GRV: 31 (39.7%) patients developed constipation, 18 (23.1%) patients developed vomiting, 14 (17.9%) patients developed abdominal distension. With regards to prevalence by occurrence, 32/78 (41%) of patients presented with two GI complications, followed by 21 patients (27%) who presented with a single GIC. Only 11 patients (14%) presented with more than three complications. Median length of stay was higher in patients with GIC (8 days) than with those who did not develop GIC (4 days). The frequency of gastrointestinal complications was significantly higher in children receiving mechanical ventilation, on sedatives and relaxants and those with multiorgan dysfunction syndrome (MODS) and inotropes

**Conclusion:**

GI complications are a frequent occurrence in the PICU and are associated with worse clinical outcomes. The use of sedative drugs and the presence of shock with MODS were amongst the important contributing factors.

## INTRODUCTION

Gastrointestinal complications (GIC) are common in critically ill children.^1^ GIC are commonly observed, as either a primary reason for admission or as a part of multiple organ dysfunction syndrome (MODS) in children admitted in the Pediatric Intensive Care Unit (PICU). Despite its prominence in critically ill patients with MODS, GIC are not included in any of the scoring systems used to assess organ failure in critical illness. A critically ill patient may develop GIC throughout his illness, e.g., high gastric residual volumes (GRVs), constipation, or vomiting. GIC has also however has been associated with high morbidity in critically ill children.^2^

GIC are often ignored in PICU which often delays enteral nutrition preventing patients from getting adequate calorie and protein intake, ultimately leading to acquired malnutrition in these patients. The lack of a uniform standard definition of GIC adds to delays in its recognition.^3^,^4^ Critical illness can result in intestinal mucosal ischemia, that further damages the gut barrier function.^5^ Recently with the increasing awareness of GIC in critically ill patients, the Working Group on Abdominal Problems (WGAP) of the European Society of Intensive Care Medicine (ESICM) proposed a set of definitions of acute gastrointestinal injury (AGI) in critical illnesses in adults for both clinical and research purposes.^2^ However, there is no such definition available for the pediatric population. Also, the associations between AGI grade, the severity of GI dysfunction, and adverse outcome remains to be elucidated.

Reintam et al reported an incidence of GIC of 59% in their mixed-ICU population.^5^ Common GIC included vomiting, high gastric residual volume (GRV), bowel distension, diarrhea, and GI bleeding, in their report. There is limited published literature on the frequency and outcome of GIC related to enteral feeding in critically ill children.^5^,^6^ Critically ill patients with GIC have a prolonged length of stay in ICU and higher mortality as compared to those without gastrointestinal complications.^2^,^5^,^6^,^7^

The epidemiological data on GIC in PICU is scarce. However, our experience with sick children has shown GIC to be associated with worse patient outcomes. We, therefore, aimed to determine the frequency of GIC and its association with outcomes at our PICU.

## METHODS

After obtaining approval from the Institutional Ethical Review Committee (Ref# 3548-Ped-ERC-15, Dated May 20, 2020), this descriptive study was prospectively conducted in The Pediatric ICU of The Aga Khan University Hospital, Karachi from September 2015 to January 2017. Ours is a multi-disciplinary eight bedded PICU, with three trained intensivists providing 24-hour coverage.

Based on a study by Lopez-Herce et al, that reported a frequency of GIC in critically ill children to be 11.5%^3^ with a margin of error of 5%, a power (1-β) of 80%, 190 patients were included in this study by using nonprobability, consecutive sampling technique. We routinely start enteral feeding through a nasogastric tube within 4-8 hours after admission/resuscitation unless contraindicated for enteral feeding or nasogastric tube (NGT). The continuous feeding is preferred in patients who are receiving vasoactive drugs.

A nutrition protocol (see annex) was used to initiate and monitor feeding in our patients. GRV was considered to be high when it exceeded half the volume of the previous feed given. GRV is assessed every four hours after ICU admission for a total of six times per day. Children admitted primarily with gastrointestinal symptoms like GI Bleeding or Acute Pancreatitis, Status-Post laparotomy, and those who stayed less than 24 hours in the PICU were excluded. Gastrointestinal complications were defined as the presence of at least one of the following pre-defined gastrointestinal problems in patients during their first-week of PICU stay as shown in Table-I.^3^ All GIC were recorded, including the number of episodes and day of presentation.

**Table-I:** Definition of Gastrointestinal Complications (GIC).

Vomiting	Involuntary, forceful expulsion of the contents of one’s stomach
High Gastric Residue	More than 50% of feed aspirated in previous 4 hour OR more than two-hour volume aspirated of the continuous feed being administered
Diarrhea	More than 4 stools per day or change in the consistency of stool
Constipation	No stool for more than 48 hours
GI bleeding	Presence of macroscopic blood in vomitus, nasogastric aspirates or stool
Abdominal Distension	Visibly distended abdomen and/OR Increase in abdominal girth by 2 cm from the baseline OR X-ray abdomen shows significantly dilated bowel loops

The primary endpoint was defined as the frequency of gastrointestinal complications developed in critically ill children during the first-week in PICU. Secondary outcomes included the association of GIC to mortality, malnutrition, length of PICU stay, and the risk factors for the development of GIC. A structured data collection form was used for data collection, including demographic data, clinical variables, including admitting diagnosis, PRISM-III score for severity assessment,^8^ use of PICU therapies like mechanical ventilation and inotropes, presence of MODS according to IPSCC 2005^7^ as well as predefined GICs (as shown in Table-I) and hospital discharge as alive or deceased.

All data were entered and analyzed using Statistical packages for social science version 22 (SPSS Inc., Chicago, IL). The quantitative variables like age, weight, height, length of stay, and duration of mechanical ventilation are expressed as mean with standard deviation and qualitative variables are expressed as percentages or range. T-test and chi-square tests were used for continuous and categorical variables respectively. Binary logistic regression analysis was performed for the assessment of factors associated with the development of GI complications. P-value ≤ 0.05 was considered as significant.

## RESULTS

From a total of 448 patients screened for eligibility, 190 patients met inclusion criteria and 78 (41%) developed one or more GIC during the first week of their PICU admission. The GIC have been defined in Table-I. The median age of the study population was 43 months (range 1m-18years, interquartile range= 78). Our study population consisted of 101 (53.5%) males and 89 (56.8%) females with a weight mean of weight of 10.2 kg (range 3.6–68 kg)

The overall median PRISM score of the study population was 6 [IQR (4-10)]. 78 patients (41%) developed GIC during the study period. Of those, 27 (34.6%) were admitted for CNS disorders, 19 (24.4%) were admitted for miscellaneous disorders, 14 (17.9%) for cardiovascular disorders (Congenital heart disease, repaired or unrepaired, cardiomyopathies, myocarditis), followed by 7.6% of children with a respiratory illness. Only 4 (5.13%) were surgical patients. 36/78 (46.1%) with MODS developed GIC while 68/78 (87.1%) with GIC were mechanically ventilated. The length of stay was higher in patients with GIC (8 days) than with those who did not develop GIC (4 days). Patients who developed GIC had a median PRISM-III score of 8. Among ICU therapies, 77% of patients in the GIC group and 66% of patients in the non-GIC group required vasoactive inotropic support (p=0.003). Sedatives including nalbuphine, and/or dexmedetomidine and neuromuscular blocking agents like atracurium and cisatracurium were prescribed in 68% of the patients with non-GIC patients requiring it more that the GIC group. Of the patients who expired, none were due to GIC; However, shock with MODS was the major underlying diagnosis (Table-II).

**Table-II:** Demographic and Clinical Profile of Patients.

Variables	All (n=190) (%)	GICP^*^ (n=78) (%)	GICN^#^ (n=112) (%)	p-value
Age in months (median) IQ range	43 (39-82)	62 (96)	35 (78)	0.11
***Gender***				
Male	101 (53.2)	43 (55.2)	58 (51.8)	0.64
Female	89 (46.8)	35 (44.8)	54 (48.2)	
PRISM-III score, Median (IQR)	6 (4-10)	8 (4-12)	5 (3-7)	0.002
***Diagnostic Category:***				
Central Nervous System	52 (27)	27 (34.6)	25 (22.3)	
Respiratory System	39 (21)	06 (7.69)	33 (29.2)	
Cardiovascular System	26 (14)	14 (17.9)	12 (10.7)	0.41
Surgery	16 (8.4)	04 (5.13)	12 (10.7)	
Trauma	12 (6.3)	08 (10.3)	04 (3.9)	
Miscellaneous ^@^	45(23.3)	19 (24.4)	26 (23.2)	
Co-morbidity	40 (21.1)	18 (23.0)	22 (19.6)	0.57
Use of Mechanical Ventilation	139 (73.1)	68 (87.1)	71 (63.3)	<0.001
Shock including use of inotropes	133 (70)	59(77.7)	74 (66.6)	0.087
Use of sedatives	130 (68.4)	63 (80.76)	67 (59.8)	0.002
Use of paralytics	128 (67.3)	60 (76.9)	67 (59.8)	0.014
Outcome (deceased)	10 (5.3)	07 (8.97)	03 (2.67)	0.056
Length of stay (Day) Median (IQR)	12 (8-16)	8 (6-10)	4 (3-8)	<0.001

* Gastrointestinal Complications,

# No gastrointestinal complications, ^Standard deviation, $Multiorgan Dysfunction syndrome,

@ Sepsis/Hematology/Oncology/Near drowning/Poisoning/DKA/Tetanus/Infections/Autoimmune.

**Fig.1:**
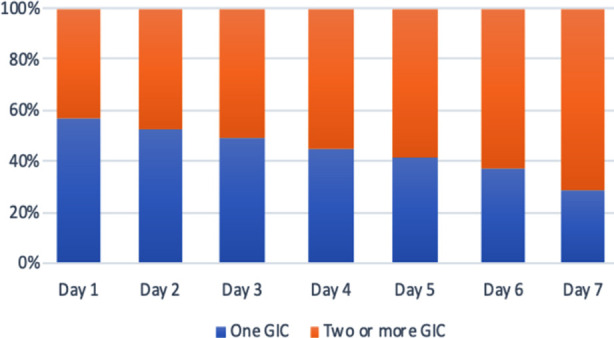
The prevalence of gastrointestinal (GI) symptoms daily during the first week in intensive care unit (ICU)

GIC developed within the first 48 hours of admission in 78 (41%) patients. Of the patients who developed GIC, 37 (47.4%) patients developed high GRV: 31 (39.7%) patients developed constipation, 18 (23.1%) patients developed vomiting, 14 (17.9%) patients developed abdominal distension. With regards to prevalence by occurrence, 32/78 (41%) of patients presented with two GI complications, followed by 21 patients (27%) who presented with a single GIC. Only 11 patients (14%) presented with more than three complications. The Prevalence of daily gastrointestinal symptoms during the first week in PICU (Fig.1).

On multivariate logistic regression analysis, the odds of having GI complications was 2.34 times higher in children receiving sedatives like nalbuphine, and/or dexmedetomidine during PICU stay. Both sedative and or relaxant use (p-value -0.010) and shock with MODS (p-value -0.002) have a statistically significant association with GIC (Table-III).

**Table-III:** Multivariable logistic regression analysis of factors determining the occurrence of GI complications.

Variable	Exp of β (Prevalence ratio)	95 % CI	p-value
Use of Sedatives	2.34	1.22, 4.50	0.001
Shock with MODS	0.48	0.22, 0.70	0.002

## DISCUSSION

We found that 41% of our critically ill children had GIC during the first week of their PICU stay which is higher than that shown by Reintem (16%) in their adult ICU patients.^6^ Adult colleagues have reported the frequency of GIC up to 60%.^2^,^6^,^9^ Our results are much higher than what has been reported in the literature; an incidence of 11.5%.^3^ The reason for such variability can be attributed to the lack of existing diagnostic criteria for GIC in both the adult and pediatric populations. Also, the fact that limited literature existing on gastrointestinal complications in critically ill children has been reported in specific populations, e.g., by Martinez et al^10^ [mechanically ventilated children] and another study from Spain^3^ in children on transpyloric nutrition. Another possible reason for the higher frequency of GIC in our population could be the inclusion of all children, regardless of the type of feeding or ventilation status.

Twenty seven (34.6%) of our patients with GIC had an underlying CNS condition. This is consistent with studies,^3^ where neurological conditions have been attributed as the three most commonly occurring diagnoses.^3^,^10^ Gastrointestinal dysfunction is amongst the common physiologic complications associated with traumatic brain injury.^10^ This feeding intolerance in particular may present with vomiting and abdominal distension.^11^ Mechanisms proposed to include impaired gastrointestinal motility, gastroesophageal reflux, delayed gastric emptying that may be related to reduced lower esophageal sphincter tone.^12^,^13^

We saw a high percentage of 37/78 (47%) of patients with high gastric residual volumes (HGRVs) in our cohort. Similar results have previously been reported in literature.^9^,^14^ A randomized controlled trial by Horn et al from Brisbane defines a high GRV greater than 5ml/kg in a group of critically ill pediatric patients.^15^ High GRVs have been associated with interruptions in enteral feeding, thereby contributing to undernutrition in the PICU.^10^ In fact, on a similar note, the routine measurement of GRVs has been questioned as a tool to assess feeding intolerance. Although GRVs were reported to be the most frequently observed (47.4%) complication in our cohort, it is imperative to define absolute criteria with regards to enteral feeding in critically ill children, and not solely base decision on one parameter. Interestingly, an observational study from Europe^16^ states that not routinely measuring gastric residual volume as opposed to a routine measure of GRVs did not increase the incidence of ventilator acquired pneumonia, aspiration, or vomiting. These results offer food for thought and therefore we recommend the use of a standard feeding protocol to guide practice. Larger multicenter trials are required to provide clinicians with further evidence.

We found a significant association of the development of GIC with the use of sedative drugs like nalbuphine. Sedatives and muscle relaxants have been shown to affect enteral nutrition as they reduce gastrointestinal motility.^17^ Lopez-Herce et al have also mentioned sedatives and relaxants as an important reason for gastrointestinal morbidity in children.^18^ Our results show that enteral nutrition (EN) can be used in critically ill children despite being on sedatives and muscle relaxants, although constipation and abdominal distention must be carefully monitored in children especially those receiving continuous infusions of sedatives and relaxants.

In our study, shock was one of the most important risk factors for gastrointestinal complications. Enteral nutrition has been shown to increase splanchnic metabolic demands, which increase manifolds in states of sepsis and shock and may lead to altered tolerance and gastrointestinal complications.^14^ It has been advised to advance enteral nutrition with caution in critically ill children.

The critically ill children receiving mechanical ventilation were at high risk for developing GIC in our PICU. Mutlu et al narrated the various spectrums of GIC in critically ill patients receiving mechanical ventilation.^14^ The integration of GIC with MODS in critically ill children perpetuates mortality in our cohort and Reintam et al had similar observation.^5^.^6^

The benefits of a protocolized or guideline-driven nutrition delivery strategy have been described in previous studies.^19^-^21^ Our current study reinforces the role of uniform guidelines in improving bedside nutrient delivery during critical illness. The use of EN algorithms/protocols has been associated with decreased time to initiation of EN, increased EN delivery and decreased reliance on PN, and increased likelihood of achieving nutrient delivery goals.

## CONCLUSIONS

GI complications are a frequent occurrence in the PICU and are associated with worse clinical outcomes. High gastric residual volume was the most common GIC. The use of sedative drugs and presence of shock with MODS were important factors for GIC. We recommend multi-center studies and algorithmic protocol-driven approach to enteral nutrition for critically ill children.

### Authors Contribution:

**SI & MS:** Conceived, designed and did statistical analysis & editing of manuscript, is responsible for integrity of the study.

**AL & AHQ:** Reviewed and approved the final manuscript and helped with manuscript writing.
